# Deactivation of ATP-Binding Cassette Transporters ABCB1 and ABCC1 Does Not Influence Post-ischemic Neurological Deficits, Secondary Neurodegeneration and Neurogenesis, but Induces Subtle Microglial Morphological Changes

**DOI:** 10.3389/fncel.2019.00412

**Published:** 2019-09-12

**Authors:** Daniel Manrique-Castano, Maryam Sardari, Tayana Silva de Carvalho, Thorsten R. Doeppner, Aurel Popa-Wagner, Christoph Kleinschnitz, Andrew Chan, Dirk M. Hermann

**Affiliations:** ^1^Department of Neurology, University Hospital Essen, University of Duisburg-Essen, Essen, Germany; ^2^Department of Neurology, University Medicine Göttingen, Göttingen, Germany; ^3^Center of Experimental and Clinical Medicine, University of Medicine and Pharmacy, Craiova, Romania; ^4^Department of Neurology, Inselspital, Bern University Hospital, University of Bern, Bern, Switzerland

**Keywords:** focal cerebral ischemia, microglial morphology, mouse, multidrug resistance transporter, neurodegeneration, neurogenesis, neurological recovery, P-glycoprotein

## Abstract

ATP-binding cassette (ABC) transporters prevent the access of pharmacological compounds to the ischemic brain, thereby impeding the efficacy of stroke therapies. ABC transporters can be deactivated by selective inhibitors, which potently increase the brain accumulation of drugs. Concerns have been raised that long-term ABC transporter deactivation may promote neuronal degeneration and, under conditions of ischemic stroke, compromise neurological recovery. To elucidate this issue, we exposed male C57BL/6 mice to transient intraluminal middle cerebral artery occlusion (MCAO) and examined the effects of the selective ABCB1 inhibitor tariquidar (8 mg/kg/day) or ABCC1 inhibitor MK-571 (10 mg/kg/day), which were administered alone or in combination with each other over up to 28 days, on neurological recovery and brain injury. Mice were sacrificed after 14, 28, or 56 days. The Clark score, RotaRod, tight rope, and open field tests revealed reproducible motor-coordination deficits in mice exposed to intraluminal MCAO, which were not influenced by ABCB1, ABCC1, or combined ABCB1 and ABCC1 deactivation. Brain volume, striatum volume, and corpus callosum thickness were not altered by ABCB1, ABCC1 or ABCB1, and ABCC1 inhibitors. Similarly, neuronal survival and reactive astrogliosis, evaluated by NeuN and GFAP immunohistochemistry in the ischemic striatum, were unchanged. Iba1 immunohistochemistry revealed no changes of the overall density of activated microglia in the ischemic striatum of ABC transporter inhibitor treated mice, but subtle changes of microglial morphology, that is, reduced microglial cell volume by ABCB1 deactivation after 14 and 28 days and reduced microglial ramification by ABCB1, ABCC1 and combined ABCB1 and ABCC1 deactivation after 56 days. Endogenous neurogenesis, assessed by BrdU incorporation analysis, was not influenced by ABCB1, ABCC1 or combined ABCB1 and ABCC1 deactivation. Taken together, this study could not detect any exacerbation of neurological deficits or brain injury after long-term ABC transporter deactivation in this preclinical stroke model.

## Introduction

Despite considerable progress in acute stroke treatment, i.e., intravenous thrombolysis and mechanical thrombectomy, stroke remains the leading cause of long-term disability. Major efforts have been made establishing pharmacological neuroprotection therapies, largely without success. A major obstacle for pharmacological compounds is the blood-brain barrier, which actively prevents drug brain entry. The blood-brain barrier contains ATP-binding cassette (ABC) transporters, which bind lipophilic xenobiotics with high affinity, among which are many brain-penetrating drugs (Soontornmalai et al., 2006; Hermann and Bassetti, 2007). Upon ischemia, ABC transporters exhibit coordinated expression changes on brain endothelial cells that impede drug brain accumulation. Thus, the ABC transporter ABCB1, which is preferentially expressed on the luminal endothelial membrane and carries drugs in direction from the brain to blood, is upregulated (Spudich et al., 2006), whereas ABCC1, which is preferentially expressed on the abluminal endothelial membrane and carries drugs in the opposite direction from the blood to brain, is downregulated after intraluminal middle cerebral artery occlusion (MCAO) (Kilic et al., 2008). This coordinated regulation was shown to persist over up to 24 h post-MCAO and then returned to basal levels (Spudich et al., 2006; Kilic et al., 2008). We previously showed that the presence and absence of ABC transporters influences drug concentrations in the ischemic brain by up to an order of magnitude or even more, thus modifying neuroprotective drug efficacy (Spudich et al., 2006; Kilic et al., 2008; ElAli and Hermann, 2010). In view of the strong impact of ABC transporter blockers, the pharmaceutical industry has developed clinically applicable inhibitors, which have been used in phase 2 and 3 trials (Robey et al., 2018). In models of Alzheimer’s disease, concerns have been raised that the long-term inhibition of ABCB1 and ABCC1 may exacerbate neurodegeneration as a consequence of reduced β-amyloid clearance (Cirrito et al., 2005; Krohn et al., 2011). In focal cerebral ischemia, we did not notice any aggravation of brain injury following ABCB1 or ABCC1 deactivation in the acute stroke phase, i.e., during the first 72 h post-MCAO (Spudich et al., 2006; Kilic et al., 2008). This observation did not rule out the exacerbation of brain injury in the subsequent post-acute phase, where major efforts are currently made establishing neurorestorative treatments. To explore possible injury-promoting effects, we herein studied consequences of a long-term delivery of the ABCB1 and ABCC1 inhibitors tariquidar and MK-571 on post-ischemic neurological deficits, delayed neurodegeneration and brain tissue remodeling in mice exposed to intraluminal MCAO.

## Materials and Methods

### Legal Requirements and Animal Housing

Experiments were conducted with government approval (G1585/16) according to EU guidelines (Directive 2010/63/EU) for the care and use of laboratory animals. Animals were housed in groups in a 12 h:12 h light/dark cycle.

### Statistical Planning, Blinding, and Randomization

Statistical planning for the neurological examinations assumed an alpha error of 5% and beta error (1–statistical power) of 20%, for which 18 animals/group were required (Wang et al., 2018). Experimenters were blinded by a third person providing drugs with dummy names. Animal randomization was done by using an open access R code.

### Experimental Procedures and Neurological Tests

Male C57BL/6 mice (23–27 g, Harlan, Horst, Netherlands) were exposed to 30 min left-sided intraluminal MCAO during 1.5% isoflurane anesthesia (30% O_2_, remainder N_2_O) (Wang et al., 2018). We decided to use a model of comparably mild focal cerebral ischemia associated with moderate neurological deficits, since we hypothesized that ABC transporter deactivation would increase neurological deficits. Hence, a model was required, in which an augmentation of deficits could reliably be detected. Rectal temperature was kept at 37.0°C using a feedback-controlled heating system. Laser Doppler flow (LDF) was monitored by a flexible probe above the core of the middle cerebral artery territory. Immediately after reperfusion, animals were intraperitoneally treated for 4 weeks with vehicle (0.9% NaCl containing 4% DMSO), tariquidar (8 mg/kg/d; Sigma, Deisenhofen, Germany), MK-571 (10 mg/kg/d; Enzo, Lörrach, Germany), or tariquidar (8 mg/kg/d) plus MK-571 (10 mg/kg), as described before (Spudich et al., 2006; Kilic et al., 2008). Neurological deficits were evaluated weekly in the animals’ dark cycle using Clark’s neurological score (Clark et al., 1997), RotaRod, tight rope and open field tests (Bacigaluppi et al., 2009; Wang et al., 2018). Animals were sacrificed 14 (8 animals/group), 28 (8 animals/group) or 56 (18 animals/group) days post-MCAO by transcardiac perfusion with ice-cold 0.9% NaCl followed by 4% paraformaldehyde in 0.9% NaCl. In animals sacrificed at 14 days post-MCAO, bromodeoxyuridine (BrdU; 50 mg/kg) was intraperitoneally (i.p.) delivered daily at 7–13 days (Wang et al., 2018). In case of animal dropouts, missing animals were replaced by new animals. Dropout rates were 10 out of 44 animals operated (22.7%) in the vehicle group, 15 out of 49 animals (30.6%) in the tariquidar group, 15 out of 50 animals (30.0%) in the MK-571 group and 6 out of 41 animals (14.6%) in the tariquidar plus MK-571 group, respectively.

Sham-operated mice were prepared by exposing mice to 1.5% isoflurane anesthesia (30% O_2_, remainder N_2_O). A midline neck incision was made, and the left-sided carotid arteries were isolated but left intact. These animals received intraperitoneal vehicle injections as specified above, and were sacrificed after 14 days. In sham-operated mice, no animal dropouts were noted. Brains were cut into 20 μm coronal cryostat sections.

### Volumetry/Planimetry

Brain sections collected at millimeter intervals across the brain were stained with cresyl violet. Brain tissue was outlined. Brain volume, striatal volume, and corpus callosum thickness were determined as described (Wang et al., 2018).

### Conventional Immunohistochemistry

Sections obtained from the bregma level, i.e., the core of the middle cerebral artery territory, were stained with chicken anti-neuronal nuclei (NeuN) (1:300; ABN91 Merck-Millipore, Darmstadt, Germany), rabbit anti-ionized calcium-binding adaptor protein (Iba)-1 (1:300; 019-19741; Wako-Chemicals, Neuss, Germany) and rat anti-glial fibrillary acidic protein (GFAP) (1:300; 13-0300; Thermo Fisher Scientific, Waltham, MA, United States) antibodies. Primary antibodies were detected by secondary Alexa Fluor-488, Alexa Fluor-594, or Alexa Fluor-647 labeled antibodies. Nuclei were counterstained with Hoechst-33342 or 4′,6-diamidino-2-phenylindole (DAPI).

### Confocal Microscopy and Conventional Tissue Analysis

NeuN + neurons were evaluated using a Zeiss AxioObserver.Z1 inverted epifluorescence microscope using a 20 × objective. Iba1 and GFAP stainings were evaluated using an Axio ZoomV.16 Lens Plan-Neofluar microscope using a 1.4 × objective. Images were pre-processed and analyzed by an open-source ImageJ (National Institutes of Health, Bethesda, MD, United States) script and the pixel classification was performed using the interactive learning and segmentation toolkit Ilastik (University of Heidelberg, Heidelberg, Germany). NeuN + neurons were counted in regions of interest in the dorsolateral striatum measuring 665.6 μm × 526.5 μm using a CellProfiler (Massachusetts Institute of Technology, Cambridge, MA, United States) pipeline (information on pipeline and threshold correction available upon request). Using an open-source ImageJ script (also available upon request), the area of tissue covered by Iba1 + microglia and GFAP + astrocytes was measured. Two brain sections were examined per animal, out of which mean values were formed.

### Morphological Analysis of Activated Microglia

Sections stained with Iba1 antibody were also evaluated using a Leica SP8 confocal microscope using a 63 × objective. 20-μm-thick Z-stacks were produced that were composed of 1331.2 μm × 1331.2 μm images that were scanned at 0.5 μm steps. Following preprocessing of images, which was performed using a customized ImageJ script, a three-dimensional morphological analysis of cells was performed using the MATLAB (MathWorks, Natick, MA, United States) based script 3DMorph (York et al., 2018).

### BrdU Incorporation Analysis

In sections obtained from the bregma level, endogenous neurogenesis was examined by immunolabeling with monoclonal rat anti-BrdU (1:100; ab6326; Abcam, United Kingdom), polyclonal goat anti-doublecortin (DCX; 1:100; sc-8066, Santa Cruz, Germany) and chicken anti-NeuN (1:300; ABN91; Merck-Millipore) antibodies that were detected by Alexa Fluor-488, Alexa Fluor-594, and Alexa Fluor-647 antibodies, respectively. Nuclei were counterstained with Hoechst-33342. Sections were evaluated with a 20 × objective using an inverted AxioObserver.Z1 epifluorescence microscope (Zeiss, Jena, Germany) analyzing regions of interest containing the subventricular zone and striatum, respectively, using an open-source ImageJ script (information on threshold settings available upon request).

### Statistical Analysis

Laser Doppler flow recordings and neurological tests were analyzed by 2-way repeated measurement analysis of variance (ANOVA) followed by Tukey’s tests, histochemical data by 2-way (>2 time-points) and 1-way ANOVA (1 time-point) followed by Tukey’s tests. LDF recordings and neurological tests are presented as mean ± SD values, histochemical data as median (mean) ± interquartile range box-blots with minimum/maximum data as whiskers. Significance thresholds were set at *p* < 0.05.

## Results

### Effects of ABC Transporter Deactivation on LDF and Neurological Deficits

**FIGURE 1:**
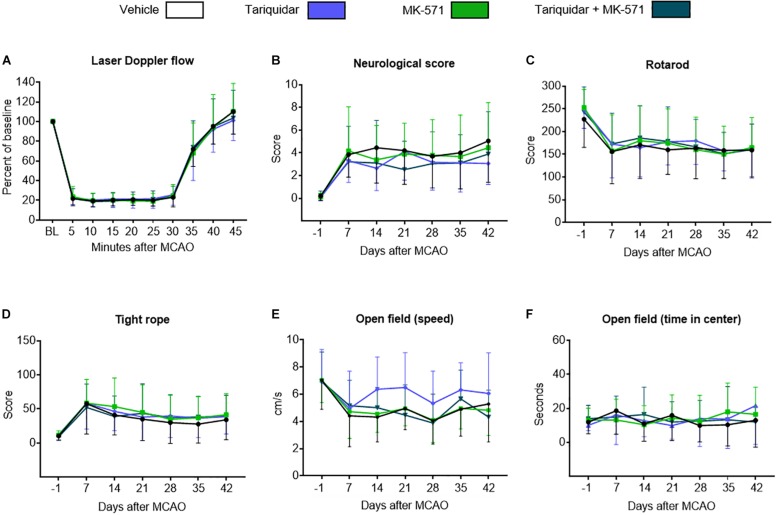
Long-term delivery of ABCB1 inhibitor tariquidar and ABCC1 inhibitor MK-571 does not compromise post-ischemic motor-coordination performance. **(A)** LDF recordings above the middle cerebral artery territory, **(B–D)** motor-coordination deficits evaluated using the Clark neurological score, RotaRod and tight rope tests, and **(E,F)** spontaneous locomotor activity, that is, speed and time in center, examined by open field tests in mice exposed to transient intraluminal MCAO. Vehicle, tariquidar (8 mg/kg/day), MK-571 (10 mg/kg/day) or tariquidar (8 mg/kg/day) plus MK-571 (10 mg/kg/day) were intraperitoneally administered over 28 days starting after reperfusion. No differences were noted between groups. Results are means ± SD values (*n* = 18 animals/group).

Intraluminal MCAO resulted in a LDF decrease to ∼20% of baseline, followed by the rapid LDF restitution after reperfusion that was not influenced by ABCB1 deactivation with tariquidar, ABCC1 deactivation with MK-571 or combined ABCB1 and ABCC1 deactivation with tariquidar and MK-571 (*F* = 0.01; *p* = 0.99) (Figure 1A). Reproducible motor-coordination deficits were noted in neurological score, RotaRod and tight rope tests (Figures 1B–D), which persisted across the observation phase and were associated with mild spontaneous motor hypoactivity in open field texts (Figures 1E,F). Motor-coordination deficits in the neurological score (*F* = 1.35; *p* = 0.98), RotaRod test (*F* = 0.14; *p* = 0.93) and tight rope test (*F* = 0.24; *p* = 0.86) were not influenced by ABC transporter deactivation. Partial reversal of spontaneous motor hypoactivity in open field tests was noted at 14–28 days in mice treated with the ABCB1 inhibitor tariquidar (Figure 1E). This effect did not reach statistical significance (*F* = 1.85; *p* = 0.14). Anxiety, evaluated by the time in the center in the open field test, was not influenced by ABC transporter deactivation (*F* = 0.89; *p* = 0.58) (Figure 1F). In order to evaluate consequences of ABC transporter deactivation under conditions of a more severe ischemia, we also performed a sensitivity analysis, in which we administered the inhibitors as above to mice exposed to 60 min MCAO, followed by 14 days survival. As for 30 min MCAO, LDF above the middle cerebral artery territory and neurological deficits evaluated by the Clark score were not altered by ABCB1, ABCC1 or combined ABCB1 and ABCC1 deactivation (Supplementary Figure S1).

### Effects on Brain Atrophy

**FIGURE 2:**
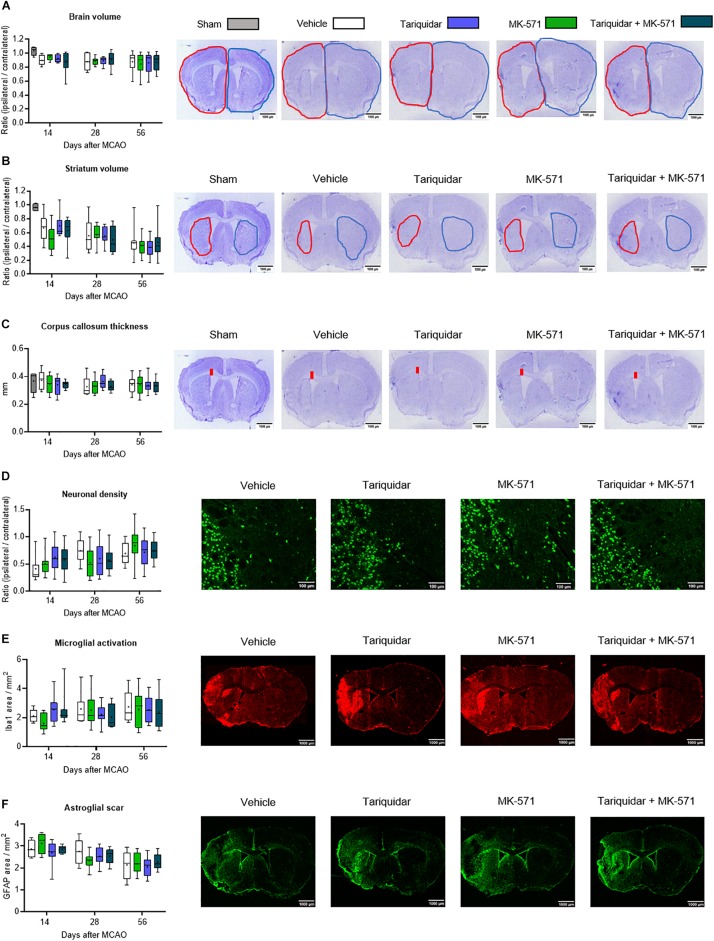
Long-term delivery of ABCB1 inhibitor tariquidar and ABCC1 inhibitor MK-571 does not influence delayed neurodegeneration and brain remodeling. **(A)** Whole brain volume, **(B)** striatal volume, and **(C)** corpus callosum thickness, outlined on cresyl violet-stained brain sections, as well as density of **(D)** NeuN + neurons, **(E)** Iba1 + microglial cells, and **(F)** GFAP + reactive astrocytes, analyzed by immunohistochemistry in the ischemic striatum. Vehicle, tariquidar (8 mg/kg/day), MK-571 (10 mg/kg/day) or tariquidar (8 mg/kg/day) plus MK-571 (10 mg/kg/day) were intraperitoneally delivered over up to 28 days post-MCAO. In **(A–C)**, sham-operated animals are also shown. In **(D–F)**, regions of interest in the dorsolateral striatum measuring 665.6 μm × 526.5 μm were evaluated. Representative microphotographs for animals sacrificed at 56 days after MCAO are shown. No differences were found between groups. Results are box-plots representing medians (lines inside boxes)/means (crosses inside boxes) ± interquartile ranges with minimum/maximum data as whiskers (*n* = 8 animals/group).

Brain injury mainly affected the ischemic striatum and most lateral parietal cortex, as described before (Wang et al., 2018). Following the acute stroke phase, in which numerous injured neurons were found throughout the middle cerebral artery territory (see Supplementary Figure S2), progressive atrophy was noted in the ischemic striatum (Figure 2A), but not cortex (not shown). Whole brain volume (*F* = 0.79; *p* = 0.49), striatum volume (*F* = 0.16; *p* = 0.91) and corpus callosum thickness (*F* = 0.33; *p* = 0.80) were not affected by the ABCB1, ABCC1, or ABCB1 and ABCC1 inhibitors (Figures 2A–C). In a sensitivity analysis in mice exposed to 60 min MCAO, brain volume, striatal volume, and corpus callosum thickness after 14 days were also unchanged (Supplementary Figure S2).

### Effects on Density of Surviving Neurons, Activated Microglial Cells and Reactive Astrocytes

Neuronal loss was prominent in the ischemic striatum (Supplementary Figure S3) and in the most lateral parietal cortex. In the ischemic striatum, the density of NeuN + surviving neurons was not influenced by ABC transporter inhibitors (*F* = 0.13; *p* = 0.93) (Figure 2D). Similarly, the density of Iba1 + activated microglia (*F* = 0.32; *p* = 0.80) and GFAP + reactive astrocytes (*F* = 0.63; *p* = 0.59) was not altered (Figures 2E,F).

### Effects on Microglial Morphology

**FIGURE 3:**
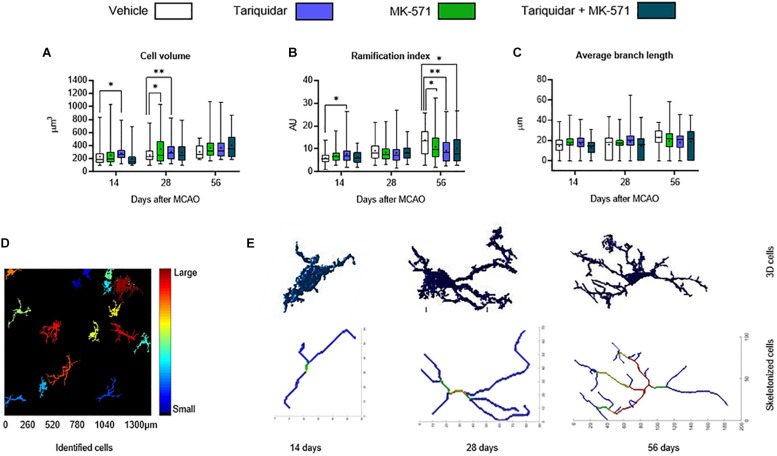
ABCB1 inhibitor tariquidar and ABCC1 inhibitor MK571 induce modest microglial morphological changes. **(A)** Cell volume, **(B)** ramification index, and **(C)** average branch length of microglial cells analyzed by Iba1 immunohistochemistry. Vehicle, tariquidar (8 mg/kg/day), MK-571 (10 mg/kg/day) or tariquidar (8 mg/kg/day) plus MK-571 (10 mg/kg/day) were intraperitoneally delivered over up to 28 days post-MCAO. **(D)** Regions of interests measuring 1331.20 μm × 1331.20 μm were evaluated in the dorsolateral striatum. Z-stacks were generated from images acquired at 0.5 μm steps. **(E)** In these Z-stacks individual cells were identified, reconstructed and analyzed. Representative cells for vehicle treated mice at all three time-points are shown. Results are box-plots representing medians (lines inside boxes)/means (crosses inside boxes) ± interquartile ranges with minimum/maximum data as whiskers (*n* = 60–80 cells/group). ^∗^*p* < 0.05/^∗∗^*p* < 0.01 compared with vehicle.

We next asked if ABC transporter deactivation might induce more mild changes of microglial morphology. In a three-dimensional analysis using the MATLAB-based script 3DMorph (York et al., 2018), microglial cell volume in the ischemic striatum was reduced by the ABCB1 inhibitor tariquidar after 14 and 28 days (that is, during drug delivery) (Figure 3A). Microglial ramification, on the other hand, was reduced by the ABCB1 inhibitor tariquidar and ABCC1 inhibitor MK-571 after 56 days (that is, after termination of drug delivery) (Figure 3B). Average microglial branch length was not influenced (Figure 3C). Our data indicate a modest microglial hyporesponsiveness during ABCB1 inhibitor treatment, and a subtle microglial overactivation after termination of ABCB1 and ABCC1 inhibitor treatments. The appearance of Z-stacks and a schematic reconstruction of representative microglial cells is shown in Figures 3D,E.

### Effects on Endogenous Neurogenesis

**FIGURE 4:**
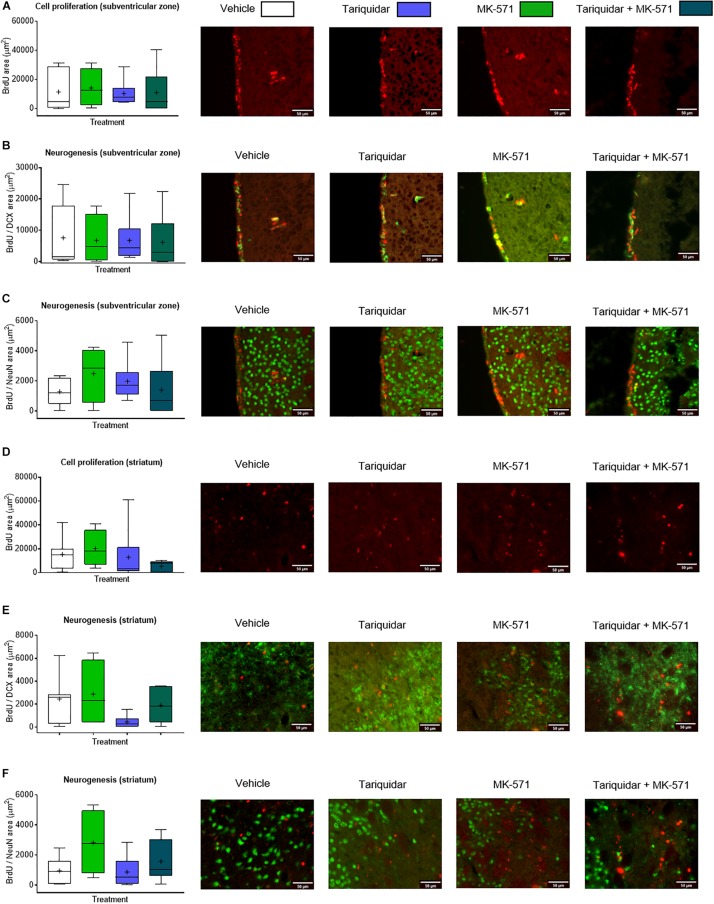
ABCB1 inhibitor tariquidar and ABCC1 inhibitor MK-571 does not influence post-ischemic endogenous neurogenesis. **(A,D)** Cell proliferation, evaluated by BrdU incorporation analysis, and **(B,C,E,F)** neurogenesis, examined by BrdU/doublecortin (DCX) and BrdU/NeuN double labeling, in mice exposed to transient MCAO in the ipsilesional subventricular zone **(A–C)** and ischemic striatum **(D–F)**. Vehicle, tariquidar (8 mg/kg/day), MK-571 (10 mg/kg/day) or tariquidar (8 mg/kg/day) plus MK-571 (10 mg/kg/day) were intraperitoneally delivered. BrdU was applied at 7–13 days post-MCAO, followed by animal sacrifice at 14 days. No differences were detected between groups. Results are box-plots representing medians (lines inside boxes)/means (crosses inside boxes) ± interquartile ranges with minimum/maximum data as whiskers (*n* = 8 animals/group).

Endogenous neurogenesis has previously been shown to be reduced in the subgranular zone of the dentate gyrus in otherwise healthy Abcb1^–/–^, but not Abcc1^–/–^ mice (Schumacher et al., 2012). As reduced neurogenesis may compromise neurological recovery (Doeppner et al., 2012), we finally evaluated whether endogenous neurogenesis was decreased in the ischemic brain of mice receiving ABC transporter inhibitors. The proliferation of endogenous neural precursor cells (NPCs) in the subventricular zone, evaluated by BrdU incorporation analysis (*F* = 0.07; *p* = 0.97), and the neuronal differentiation of NPCs in the subventricular zone, examined by BrdU incorporation analysis combined with DCX and NeuN immunohistochemistry (*F* = 0.03; *p* = 0.99 and *F* = 0.68; *p* = 0.57, respectively), was not influenced by ABCB1, ABCC1, or ABCB1 and ABCC1 deactivation (Figures 4A–C). Likewise, the proliferation and neuronal differentiation of endogenous NPCs in the ischemic striatum was unchanged (*F* = 0.18; *p* = 0.90; *F* = 1.78; *p* = 0.18 and *F* = 0.66; *p* = 0.58, respectively) (Figures 4D–F).

## Discussion

Using an intraluminal MCAO model, in which we previously characterized effects of the ABCB1 (Spudich et al., 2006) and ABCC1 (Kilic et al., 2008) inhibitors tariquidar and MK-571 on ischemic injury and the survival-promoting effects of neuroprotective drugs that are known ABC transporter substrates, we herein show that long-term ABCB1 and ABCC1 deactivation by the same inhibitors does not compromise neurological performance and delayed neurodegeneration. Only subtle changes of microglial morphology were noted in the brains of ABCB1 and ABCC1 inhibitor treated mice, whereas the overall density of Iba1 + activated microglial cells was unchanged. Astroglial scar formation and endogenous neurogenesis were not influenced by ABCB1 and ABCC1 deactivation. Already in our previous studies, we did not see any evidence for exacerbated ischemic brain injury (Spudich et al., 2006; Kilic et al., 2008). These earlier observations were confined to the first 72 h post-stroke. In the present study, we expanded these findings to the post-acute stroke phase. Tariquidar is a highly selective ABCB1 inhibitor that does not deactivate ABCC1, while MK-571 inhibits ABCC1 but not ABCB1 (Spudich et al., 2006; Kilic et al., 2008). Tariquidar has already been used in cancer trials as add-on treatment for chemotherapeutics (Binkhathlan and Lavasanifar, 2013). ABC transporter inhibitors, including ABCB1 and ABCC1 inhibitors, have already been studied in brain tumors in clinical trials (Binkhathlan and Lavasanifar, 2013). In animal models of Alzheimer’s disease, the long-term deficiency of ABCB1 and ABCC1 has previously been found to accelerate neurodegeneration as a consequence of reduced β-amyloid elimination (Cirrito et al., 2005; Krohn et al., 2011). Both ABCB1 and ABCC1 contribute to β-amyloid clearance across the blood-brain barrier.

In this study, we decided to expose young, otherwise healthy mice to focal cerebral ischemia, since we aimed to clarify how ABC transporter deactivation affects neurological recovery and brain injury independent of an associated Alzheimer’s pathology. Besides β-amyloid, ABC transporters eliminate a large number of endogenous substrates from the brain, such as glutathione, sphingolipids, nucleosides, nucleotides, cyclic nucleotides (e.g., cGMP), and glutathionized, glucuronidated and sulfated organic anions (Hermann and Bassetti, 2007), many of which have profound effects on neuronal survival and brain remodeling. We were surprised to see that ABCB1 and ABCC1 deactivation did not affect neurological recovery and secondary neurodegeneration. In our study, mild changes of microglial morphology were noted in the brains of ABC transporter inhibitor treated mice, that is, reduced microglial cell volume after 14 and 28 days (that is, during drug delivery) in mice receiving the ABCB1 inhibitor, which indicated modest microglial hyporesponsiveness, and reduced microglial ramification after 56 days (that is, after termination of drug delivery) in mice receiving the ABCB1 and ABCB1 inhibitors, which indicated subtle microglial hyperactivation. The total number of Iba1 + activated microglia was not influenced by ABCB1 or ABCC1 deactivation. In Abcb1^–/–^ mice not exposed to focal cerebral ischemia, increased microglial activation associated with hyporamification of this microglia has previously been demonstrated in the hippocampal CA3 region (Brzozowska et al., 2017). In our study, ABCB1 and ABCC1 deactivation did not influence reactive astrogliosis or endogenous neurogenesis. In Abcb1^–/–^ but not Abcc1^–/–^ mice, reduced endogenous neurogenesis revealed by DCX had previously been shown (Schumacher et al., 2012).

Strengths of our study are the adequate statistical powering and the use of a broad battery of neurological tests, which we combined with rigid structural volumetry/planimetry and immunohistochemical studies. Limitations are that we did not study the additional impact of Alzheimer’s pathology on post-ischemic neurological recovery and brain remodeling and, perhaps, that we did not evaluate middle-aged or aged mice. The brain abundance and function of ABC transporters decreases with aging (e.g., Warrington et al., 2004; Zoufal et al., 2018). We therefore did not expect an exacerbated neurodegeneration after ABC transporter deactivation in aged mice. In view of the failure of clinical trials using ABC transporter inhibitors mainly in the cancer field, research interest in these drugs has meanwhile waned (Robey et al., 2018). In view of this loss of interest, very recent studies raised the question whether ABC transporter inhibitors have prematurely been left in drug development (Robey et al., 2018). The authors argued that at the time of the discovery of existing drugs, the field lacked important knowledge about the biology of ABC transporters, e.g., their overlap in substrate binding, their species differences and their role in human brain pathologies (Robey et al., 2018). The present study could not detect any detrimental consequences of long-term ABC transporter deactivation for neurological recovery or secondary brain injury after focal cerebral ischemia. When considering further studies in ischemic stroke, detrimental consequences related to coexisting Alzheimer’s pathology should be examined.

## Data Availability

The raw data supporting the conclusions of this manuscript will be made available by the authors, without undue reservation, to any qualified researcher.

## Ethics Statement

This study was carried out in accordance with the recommendations of EU guidelines (Directive 2010/63/EU) for the care and use of laboratory animals, and approved by the Bezirksregierung Düsseldorf (G1585/16).

## Author Contributions

MS, AC, and DH designed the study. DM-C, MS, and TS performed the animal experiments. TS prepared the treatments. DM-C and MS conducted the histochemical staining and analyses. DM-C, MS, and DH analyzed the data. MS, TD, CK, AP-W, AC, and DH drafted the manuscript. All authors finalized the manuscript.

## Conflict of Interest Statement

The authors declare that the research was conducted in the absence of any commercial or financial relationships that could be construed as a potential conflict of interest.
