# Supplementary material for: Deactivation of ATP-Binding Cassette Transporters ABCB1 and ABCC1 Does Not Influence Post-ischemic Neurological Deficits, Secondary Neurodegeneration and Neurogenesis, but Induces Subtle Microglial Morphological Changes
Source: Front Cell Neurosci. 2019 Sep 12;13:412. doi: 10.3389/fncel.2019.00412 (PMC6751309; doi:10.3389/fncel.2019.00412)
Supplement: Supplementary file 1 [file Data_Sheet_1.docx]

**Supplementary Material:**

**
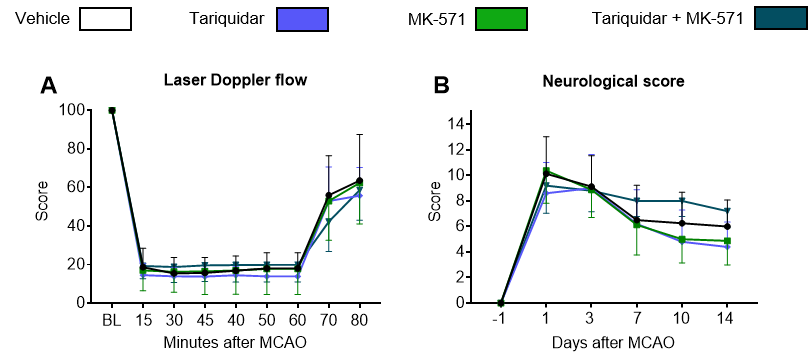
**

**Supplementary Figure S1: Long-term delivery of ABCB1 inhibitor tariquidar and ABCC1 inhibitor MK-571 do not exacerbate post-ischemic neurological deficits. (A)** LDF recordings above the middle cerebral artery territory and **(B)** neurological deficits evaluated using the Clark score in mice exposed to transient 60 minutes intraluminal middle cerebral artery occlusion (MCAO). Vehicle, tariquidar (8 mg/kg/day), MK-571 (10 mg/kg/day) or tariquidar (8 mg/kg/day) plus MK-571 (10 mg/kg/day) were intraperitoneally administered after reperfusion (protocol as main manuscript). No differences were noted between groups [F=1.82; p=0.15 and F=0.77; p=0.52, for LDF and neurological deficits, respectively]. Results are means ± SD values (n=5-8 animals/group).

**
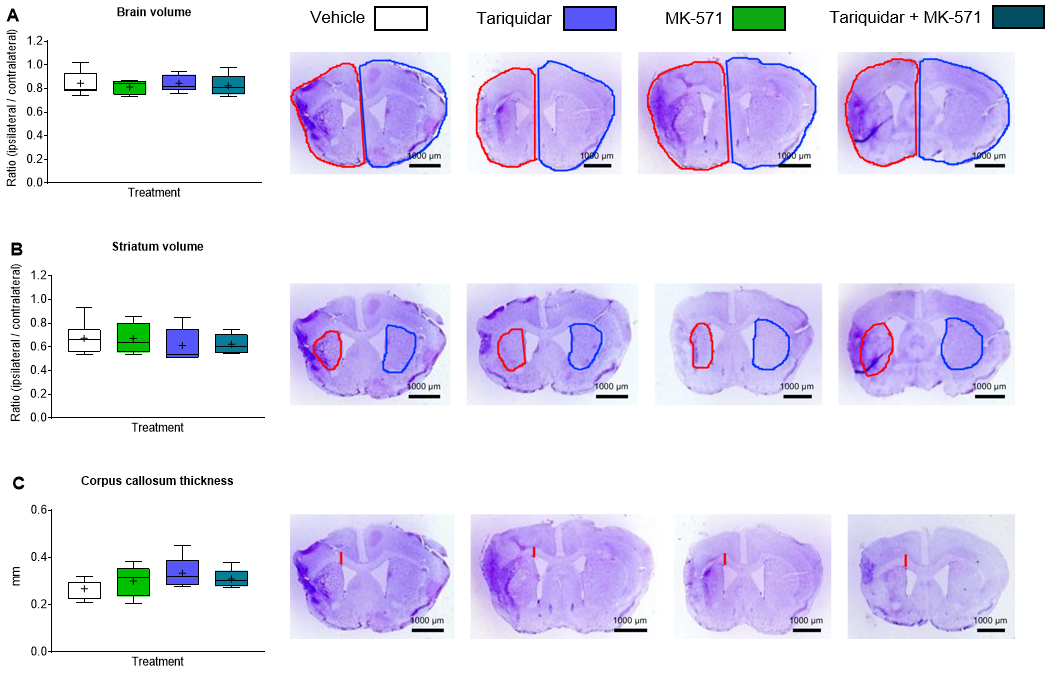
**

**Supplementary Figure S2: Long-term delivery of ABCB1 inhibitor tariquidar and ABCC1 inhibitor MK-571 does not induce delayed neurodegeneration.** **(A)** Whole brain volume, **(B)** striatal volume and **(C)** corpus callosum thickness, outlined on cresyl violet-stained brain sections in mice exposed to transient 60 minutes intraluminal MCAO. Vehicle, tariquidar (8 mg/kg/day), MK-571 (10 mg/kg/day) or tariquidar (8 mg/kg/day) plus MK-571 (10 mg/kg/day) were intraperitoneally delivered after reperfusion (protocol as in main manuscript). Representative microphotographs are also shown. No differences were found between groups [F=0.25; p=0.85, F=0.37; p=0.77, and F=1.44; p=0.25, for whole brain volume, striatal volume and corpus callosum thickness, respectively]. Results are box-plots representing medians (lines inside boxes)/ means (crosses inside boxes) ± interquartile ranges with minimum/ maximum data as whiskers (n=5-8 animals/group).


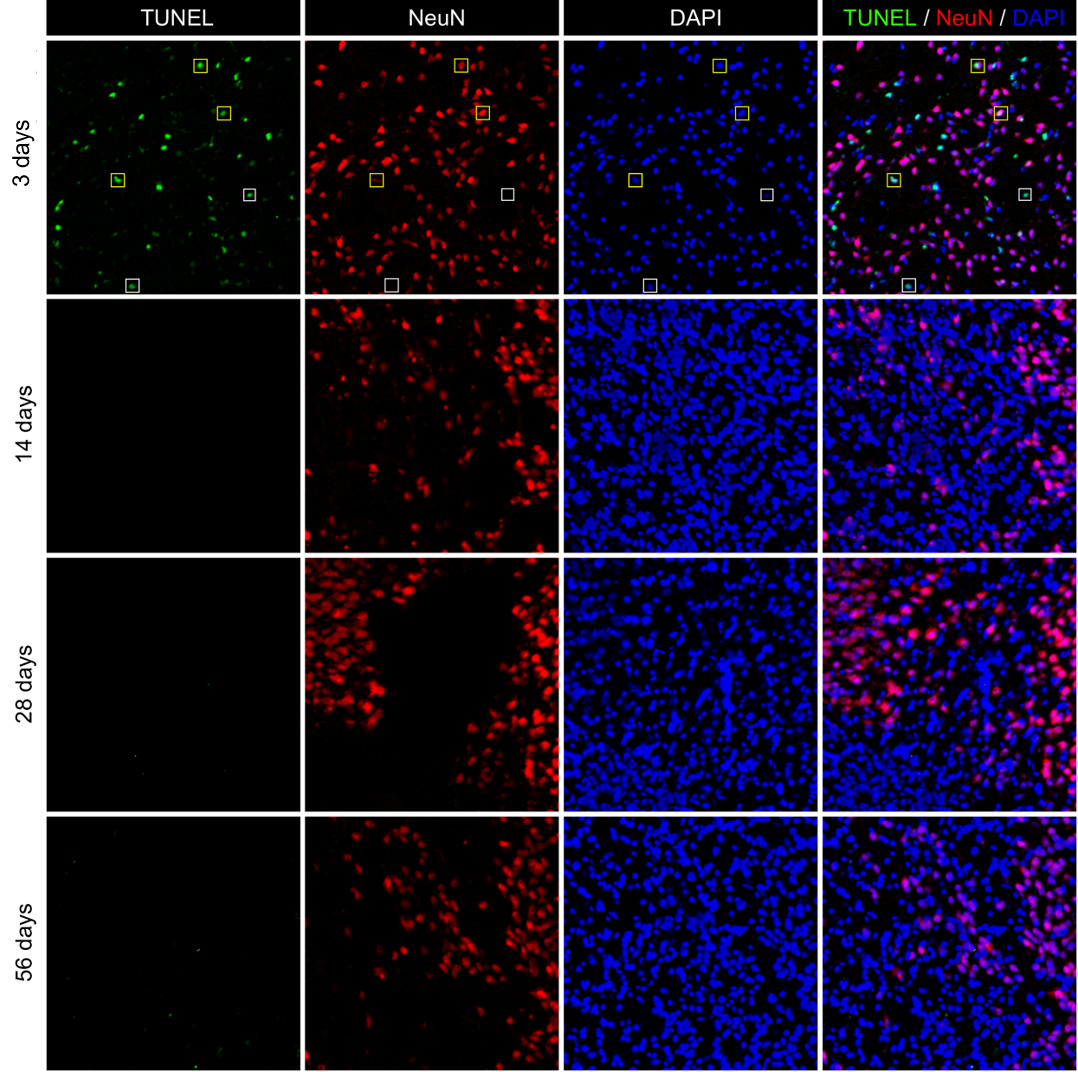


**Supplementary Figure S3: Line-up of terminal transferase-mediated nick end labeling (TUNEL)+ cells and NeuN+ neurons at different time-points after focal cerebral ischemia.** Triple staining for (from left to right) TUNEL, the neuronal marker NeuN and the nuclear marker 4',6-diamidine-2'-phenylindole (DAPI), as well as an overlay of all three stainings in the dorsolateral striatum of representative vehicle treated mice exposed to 30 minutes intraluminal MCAO followed by 3, 14, 28 or 56 days reperfusion. Note the large number of TUNEL+ cells at 3 days post MCAO. At this time-point, many TUNEL+ cells exhibit NeuN colabeling. Note the absence of TUNEL+ cells at later time-points, that is, after 14, 28 or 56 days.
